# Finite element analysis of biomechanical effects in rat tibia during tibial cortex transverse transport

**DOI:** 10.3389/fbioe.2025.1670040

**Published:** 2025-11-12

**Authors:** Puxiang Zhen, Jie Liu, Hongjie Su, Wencong Qin, Yi Ding, Shenghui Yang, Lu Wei, Ruiqing Mo, Xinyu Nie, Qikai Hua

**Affiliations:** 1 Guangxi Medical University, Nanning, China; 2 Department of Orthopaedic Surgery, Guangxi Diabetic Foot Salvage Engineering Research Center, The First Affiliated Hospital of Guangxi Medical University, Nanning, China; 3 National Demonstration Center for Experimental (General practice) Education, Hubei University of Science and Technology, Xianning, China; 4 Department of Orthopedics, The First Affiliated Hospital of USTC, Division of Life Sciences and Medicine, University of Science and Technology of China, Hefei, China

**Keywords:** tibial cortex transverse transport, diabetic rats, finite element analysis, safety, stability

## Abstract

**Objective:**

Diabetic foot ulcer (DFU) poses a major clinical burden. This study, for the first time, establishes and validates a finite element (FE) biomechanical model of tibial cortex transverse transport (TTT) in diabetic rats. By integrating micro-CT data at multiple time points, we provide a novel computational approach to assess the biomechanical safety and stability of TTT, thus bridging preclinical animal research and potential clinical translation.

**Methods:**

This study utilized a customized transverse osteotomy transport frame to establish a model of TTT for treating lower limb ischemic ulcer in diabetic rats. Postoperatively, the tibiae and fibulae Dicom were harvested by *ex-vivo* micro-CT scaning. The imaging data are processed and analyzed using mechanical analysis software by Mimics, 3-matic Medical, Geomagic Studio, Hypermesh, MSC.Patran, and MSC. Nastran to simulate the loading characteristics of the rat’s tibia and fibula with the TTT.

**Results:**

1. Peak von Mises stresses in the transport tibial bone fragment under axial compression (7.04 Mpa), axial torsion (16.91 Mpa), and three-point bending (9.40 Mpa), showed no significant differences between postoperative time points (3, 6, 9, 12, and 30 days), indicating that the overall stress change in the tibia during the tibial transverse transport process is minimal. 2. Over 8-week healing period, dynamic load sharing occurred among the transported bone fragment, original tibia, and adjustable external fixator. Progressive healing of the transported bone fragment with the surrounding bone tissue reduced the structural bearing stress of the adjustable TTT fixation. The overall stiffness of the tibia increases as the transported fragment and tibia gradually restore, further enhancing the stability of the overall tibia. 3. Under biomechanical testing conditions including axial compression, axial torsion, and three-point bending, the application of adjustable external fixators successfully repositioned free bone fragments to their anatomical alignment in the tibia without exceeding the ultimate yield strength of cortical bone tissue. Secondary fracture initiation or catastrophic structural failure was not observed during testing. The current experimental results shows the TTT fixation satisfies the required strength criteria for rat experiment.

**Conclusion:**

The TTT rat model demonstrated biomechanical stability and surgical safety *in silico*, supporting its translational potential. However, further experimental validation is required.

## 1 Introduction

Diabetic foot ulcers (DFUs) are a common and highly destructive complication of diabetes (Sa et al., 2024). Microvascular occlusion and secondary infections contribute significantly to the chronicity and non-healing nature of DFUs (Singh and Agarwal, 2025), resulting in non-traumatic lesions (Gould et al., 2021). In the late 20th century, Dr. Ilizarov of the former Soviet Union, drawing on extensive clinical practice and research, proposed the “tension-stress” rule or distraction osteogenesis principle. According to this principle, slow, continuous, and stable tissue distraction induces cellular proliferation and biosynthetic activity, leading to tissue regeneration (Ilizarov, 1989a; Ilizarov, 1989b). Distraction osteogenesis, regenerating bone post-osteotomy through distraction, exemplifies this principle (Fu et al., 2021). Widely used in limb elongation, orthopedics, and bone defect treatments, this technique addresses infections and non-unions (Sahu et al., 2024; Zhu et al., 2022). During the distraction process, bone and nearby tissues undergo significant angiogenesis, and researchers termed this phenomenon “Distraction Angiogenesis” (Kong et al., 2020; Li et al., 2021). Leveraging this, transverse tibial distraction, or Tibial cortex Transverse Transport (TTT), has effectively treated ischemic lower limb diseases like Buerger’s disease (Liu et al., 2020; Sain et al., 2023). Recently, the technique’s significant clinical effects in treating diabetic foot ulcers have garnered increasing attention (Chen et al., 2022; Chen et al., 2020; Nie et al., 2021; Qin et al., 2023). TTT, an emerging method for treating diabetic foot ulcers, is now widely used clinically. Clinical studies confirm that TTT enhances vascular regeneration in diabetic lower limbs, supporting the healing of chronic ulcers and improving blood flow in the affected areas (Hu et al., 2023). Although several animal and clinical studies have described TTT outcomes, to our knowledge, this is the first study to employ a finite element analysis (FEA) for biomechanical validation of TTT in a diabetic rat model. This work aims to fill a crucial gap in the literature by providing a rigorous computational validation that can facilitate the translation of preclinical findings to clinical practice.

Owing to limitations in clinical trials, we established a TTT therapeutic model for ischemic ulcers in diabetic rats’ lower extremities (Qin et al., 2024). This model simulates the biological effects of TTT technology and explores the mechanisms of TTT in treating diabetic foot ulcers. To confirm the success of the TTT surgical procedures in rats, we performed micro-CT scans for three-dimensional reconstruction of the rat tibia model. Using the reconstructed model, we aligned finite element mesh properties, applied loads, and set boundary and computational conditions. These steps simulate real-world stresses during normal activities of post-surgical rats, assessing the safety and stability of both the surgical model and the external TTT fixation to evaluate the modeling success.

## 2 Materials and methods

### 2.1 Surgical procedure for tibial cortex transverse transport in rats

**FIGURE 1:**
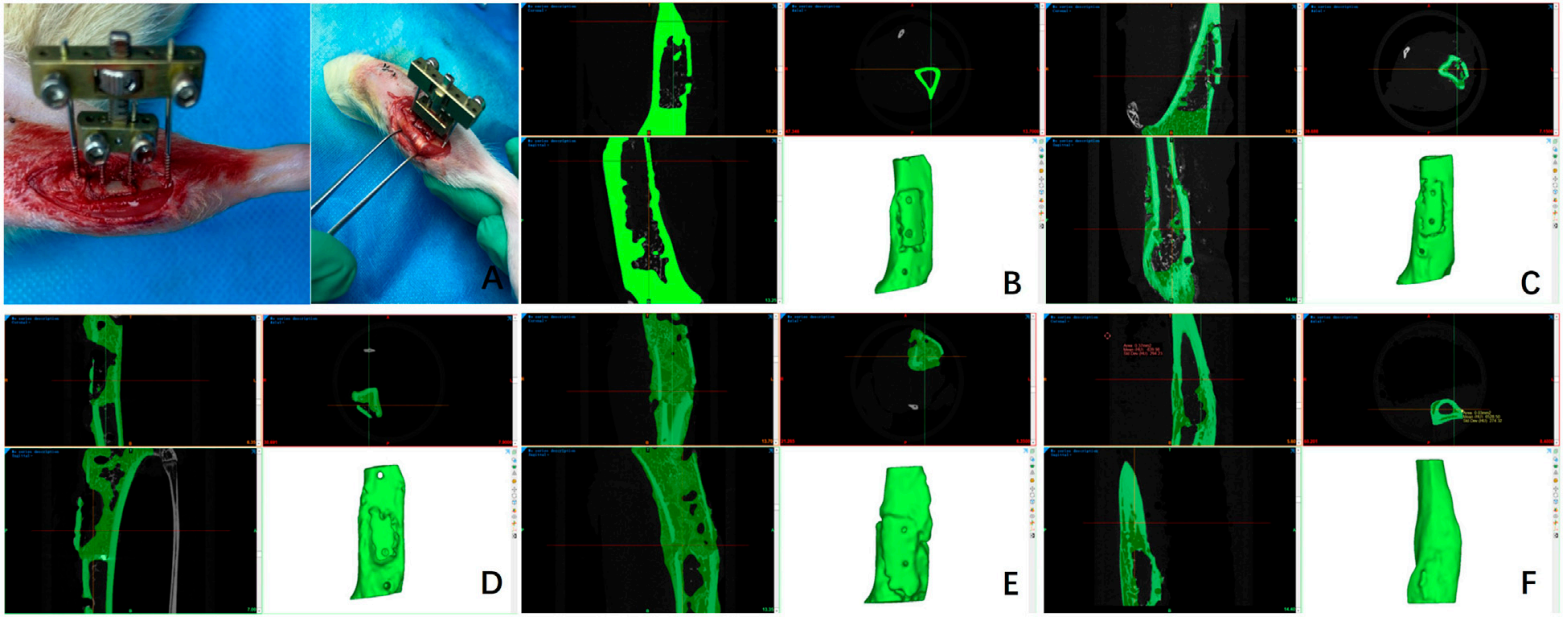
Physical diagram of the adjustable external fixator of the tibia of a rat **(A)**; Medical images of micro-CT scans of rat tibia at different times (3, 6, 9, 12, and 30days) after the free bone fragment was fixed by the external fixator **(B–F)**.

All animal procedures were approved by the Ethics Committee of Guangxi Medical University (Approval No. 2025-E0518) and complied with the ARRIVE guidelines. This study employed a customized external fixator for tibial transverse transport in rats. The fixator comprises stainless steel rods, mini screws, double-headed bolts, and hinges. Prior to surgery, rats received an intraperitoneal injection of pentobarbital (50 mg/kg) for anesthesia. A 3 cm longitudinal incision was made below the tibial tuberosity on the hind limb. The skin and muscle layers were retracted to expose the tibia. A rectangular osteotomy (8 mm × 4 mm) was performed using a 0.6 mm drill bit, centered 1 cm distal to the tibial tuberosity. Two 0.8 mm diameter pins were implanted in the center of the osteotomized segment to facilitate bone transport. 1 mm diameter pins were inserted at both ends of the osteotomy to secure the external fixator to the tibia. Subsequently, the fixator’s screws were tightened to stabilize the apparatus. Mobility of the bone fragments was maintained by adjusting the device’s nuts (Figure 1A).

### 2.2 DICOM acquisition

DICOM images of rat tibia with TTT were provided by the First Affiliated Hospital of Guangxi Medical University. Micro-CT scanning was performed with the following parameters: isotropic voxel resolution 18 μm, slice thickness 18 μm, voltage 70 kV, current 114 μA. Scans were taken at 3, 6, 9, 12, and 30 days after fixation. Prior imaging inspections and clinical evaluations confirmed the absence of bone tumors, infections, or deformities. Images were captured in anteroposterior, lateral, oblique, and dynamic positions. This provided enabling comprehensive DICOM datasets for 3D reconstruction of the rat tibia model (Figures 1B–F).

### 2.3 Constructiuon of finite element 3D models

**FIGURE 2:**
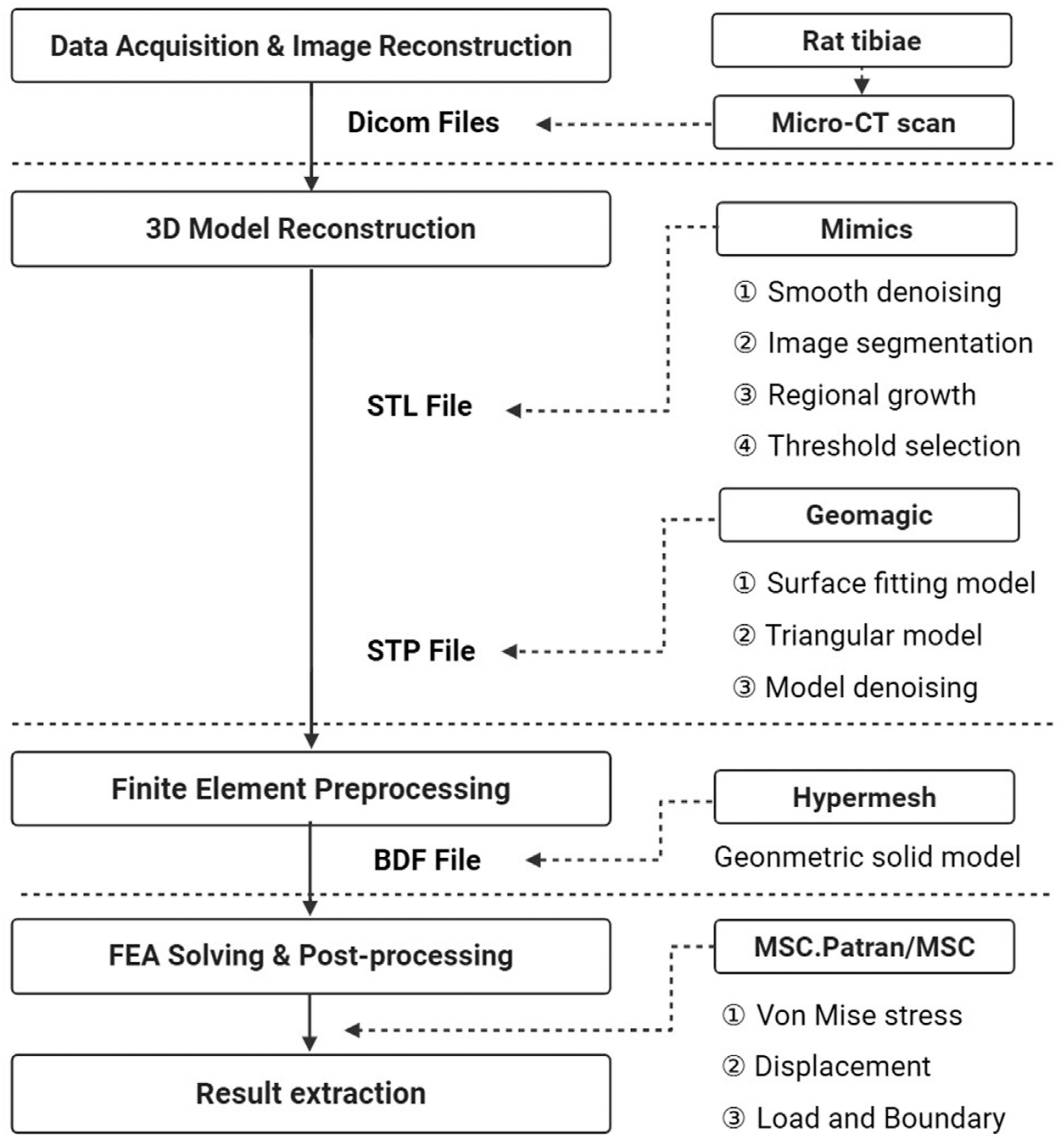
Schematic diagram of the process of 3D reconstruction and finite element technology.

The DICOM images of the rat were imported into Mimics (version 21.0, Materialise Company, Belgium) to reconstruct the three-dimensional “STL” (Stereolithography file) model at different time points. The models were then processed in 3-Matic Medical (version 13.0, Materialise Company, Belgium) and Geomagic Studio (version 2014, Raindrop Company, America) for defect repairing, noise reduction, and surface smoothing, pre paring them for finite element analysis (Figure 2).

### 2.4 Finite element mesh division

**FIGURE 3:**
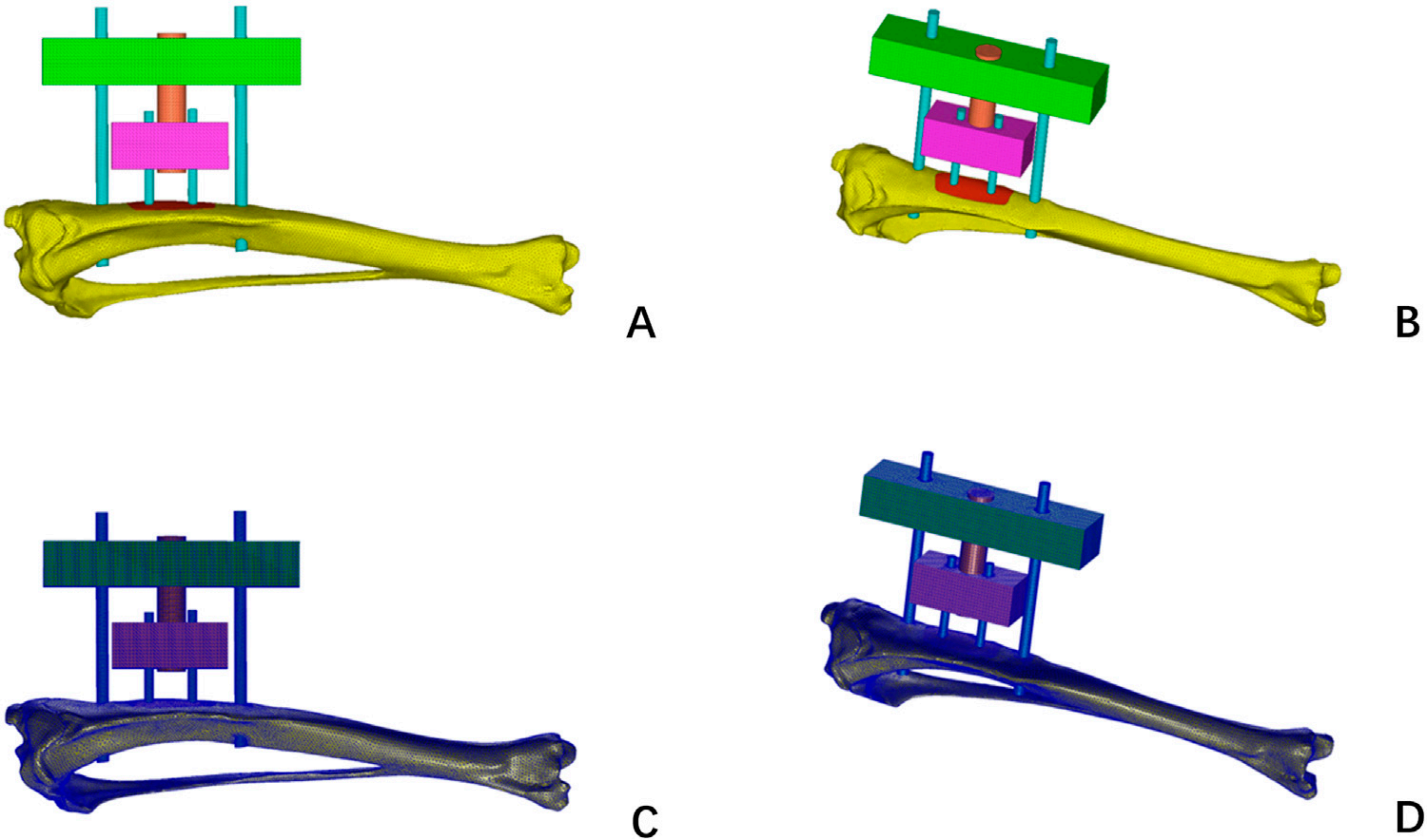
Hypermesh delineation mesh for complete rat tibia and TTT fixation **(A,B)**; Finite element mesh model diagrams of complete rat tibia and TTT fixation and extinction diagrams **(C,D)**.

The rat tibia “STP” (file conforming to STEP application) model was reverse engineered and imported into Hypermesh (version 14.0, Altair Company, America) for mesh division. The complete geometric model STP file of the rat tibia, including the adjustable external fixator, was imported into Hypermesh (version 14.0, Altair Company, America) for meshing. The meshing process involved a total of 63,727 nodes and 282,246 tetrahedral solid elements (Tet Mesh Tet4 Elements). The meshes between different bodies were set up with shared nodes to ensure the accurate transmission of forces. After the mesh division was completed, the files were exported in “BDF” (Nastran format). Based on the relationship between the density and elastic modulus in the actual image data grayscale values of the rat tibia, exported as a “BDF” file was imported back into Mimics for grayscale material parameter assignment (Figure 3).

### 2.5 Assumptions of boundary conditions

The mesh model file with completed grayscale assignment was imported into the finite element pre-processing software MSC. Patran (version 2019, MSC Software Company, America). Boundary conditions were defined: the proximal tibia was fixed at the tibial plateau with all six degrees of freedom constrained, and a cyclic axial compressive load of 1–11 N was applied to the distal tibial surface. Referencing the axial compression tests on rat tibias conducted by Bettina M. Willie (Willie et al., 2013). This approach has been previously adopted in rodent bone biomechanical studies. Human tibial loads were scaled to rats using body weight and cross-sectional area ratios, an approach validated in prior rodent biomechanics studies.This assumes linear proportionality, a limitation acknowledged in Discussion.

**FIGURE 4:**
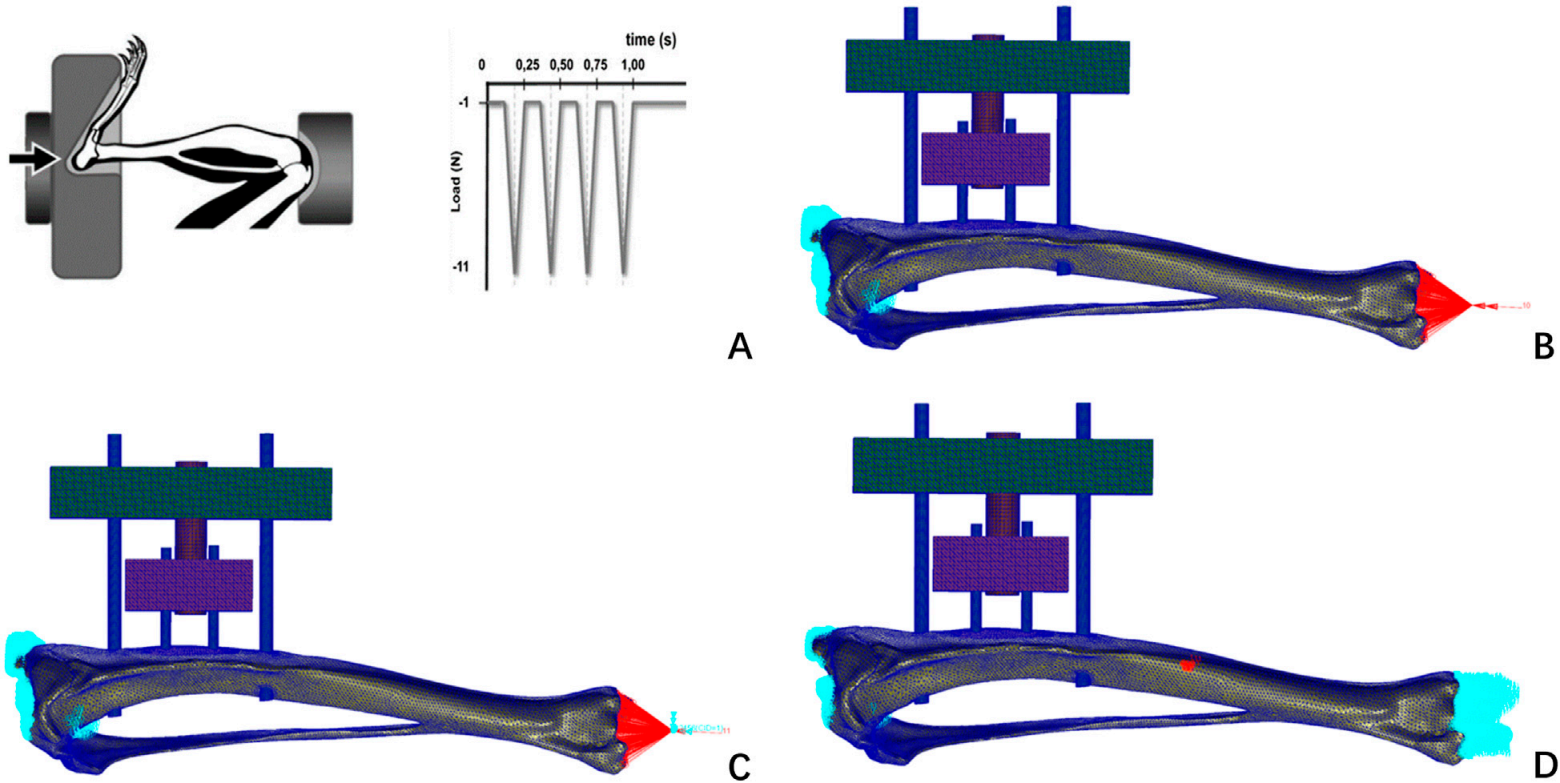
Loads and boundary constraints imposed by Bettina M. Willie’s axial compression experiments on rat tibiae **(A)**; this finite element study constrains the boundaries of a finite element model of the rat tibia **(B)** axial compression test; **(C)** axial torsion test; **(D)** three-point bending test.

Nodes near the tibial plateau were fully fixed, while some distal nodes were constrained in the X and Y directions, allowing movement only in the Z direction to ensure vertical axial compression. Additionally, biomechanical simulations of axial torsion and three-point bending were conducted (Abd Aziz et al., 2024). Lacking rat-specific load data, human tibial test loads were scaled proportionally, assuming 10 N mm for axial torsion and 5 N for three-point bending. These simulations qualitatively assessed biomechanical changes in the rat tibia with TTT fixation. Finite element analysis was performed using MSC. Nastran (version 2019, MSC Software, USA), with results visualized in its post-processing module (Figure 4).

### 2.6 Statistics analysis

All statistical analyses were performed using GraphPad Prism (8.0). Only descriptive statistics (means, ranges) were reported. No inferential statistical tests were performed due to limited sample size and the computational nature of the study.

## 3 Results

### 3.1 Biomechanical studies at different time points

**FIGURE 5:**
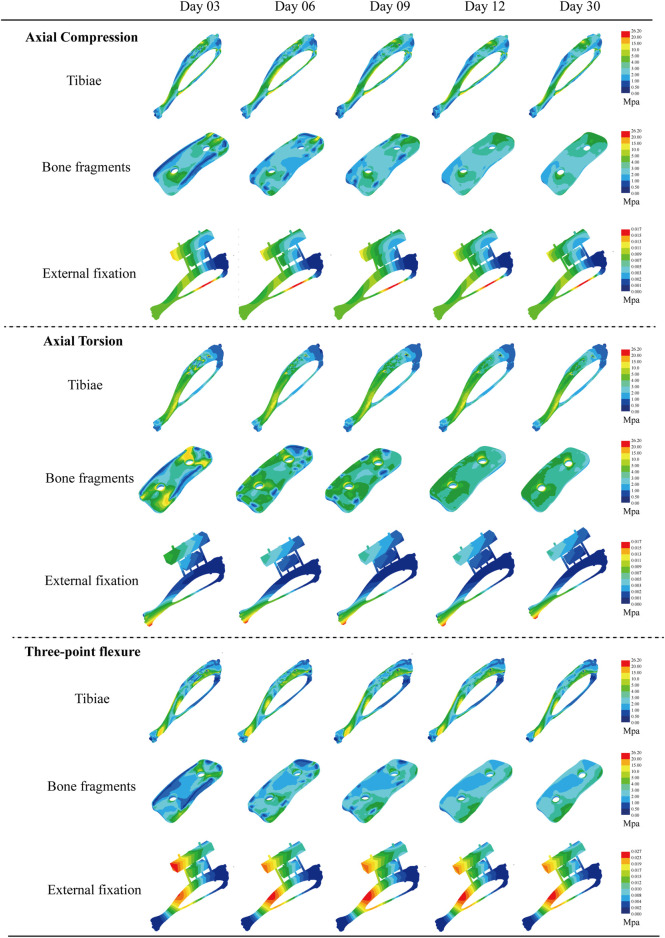
Von Mises equivalent force cloud plots of three biomechanical experiments (axial compression test, axial torsion test, and three-point bending test) performed at different moments (3, 6, 9, 12, and 30days) for each structure after external fixation of rat tibia.

Finite element simulations were conducted on a rat tibia secured by an adjustable external fixator, evaluating free bone fragments at various stages (3, 6, 9, 12, and 30 days). Simulations included three biomechanical tests: axial compression, torsion, and three-point bending. The simulations analyzed structural stresses and deformations at each time point, detailing Von Mises stress contours and total displacement of the post-fixation tibia (Figure 5).

### 3.2 Structural strength comparison

**FIGURE 6:**
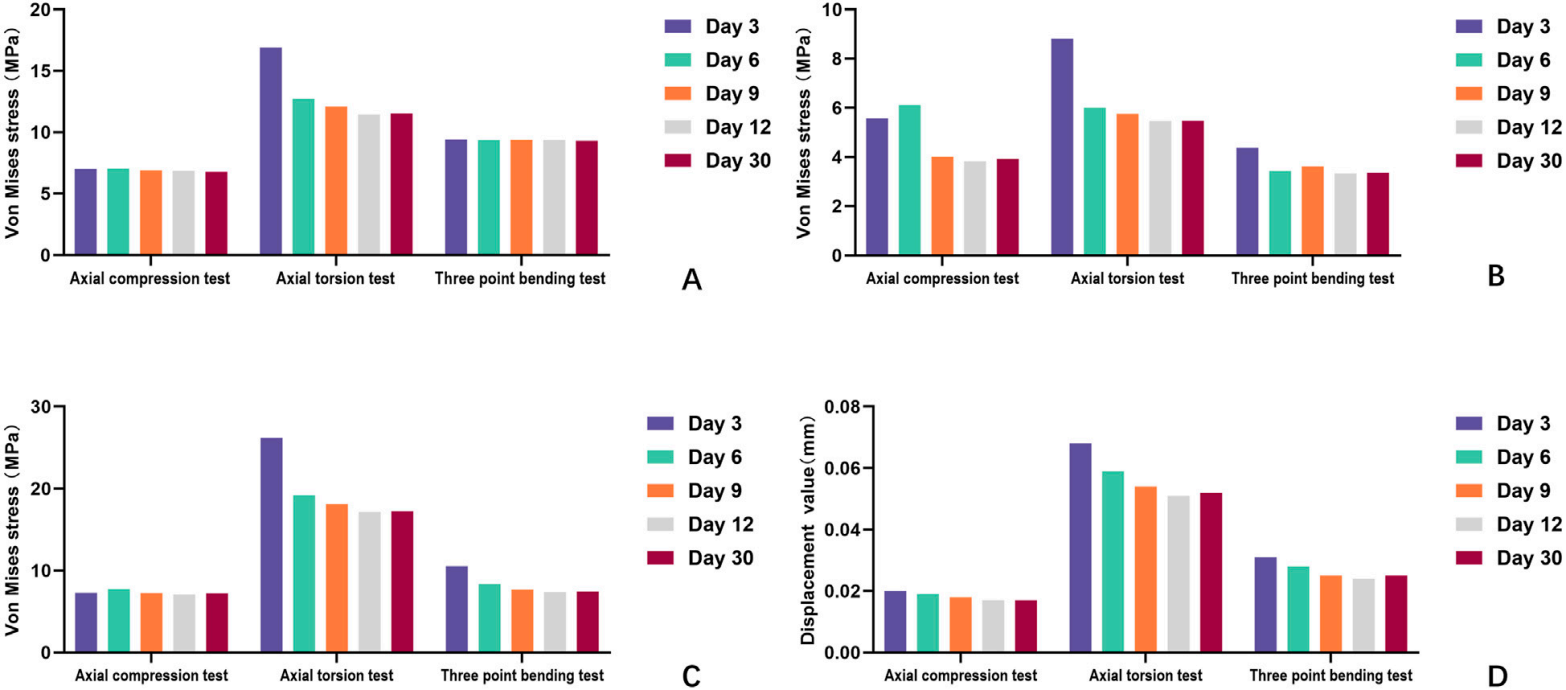
Comparison of the strength of three biomechanical tests (axial compression test, axial torsion test, and three-point bending test) of each structure at different moments (3, 6, 9, 12, and 30days) after external fixation of the rat tibia. **(A)** Comparison of peak tibial stress; **(B)** comparison of peak localized stress concentrations in free bone fragments; **(C)** comparison of peak stress in external fixation. **(D)** Comparison of overall structural deformation of the tibia.

During the experiments, we noted a distinct compressive stress area on the medial side of the rat tibia, whereas the lateral side exhibited significant tensile stress. This phenomenon mirrors the stress distribution observed in axial compression experiments on rat tibias, confirming that the modeling approach meets analytical standards. It offers a vital theoretical basis for biomechanical predictions concerning the stabilization of free bone fragments by the external fixator over time. Under axial compression (6.78–7.04 Mpa, Δ = 0.26 MPa), torsion (11.46–16.91 Mpa, Δ = 5.45Mpa), and three-point bending (9.30–9.40 Mp, Δ = 0.1 MPa), the tibia’s overall stress changes were minimal. The main effects manifested in the free bone fragments and adjacent bone tissue recovery zones. As the transported bone fragments integrated with surrounding bone tissue over time, this enhanced the tibia’s overall stiffness. At this stage, the transported bone fragments began to distribute the external forces, applied during experiments, across the original tibia and the TTT fixation, reducing stress on the latter. Throughout this process, the observed peak von Mises stress in all materials remained below their ultimate yield strengths—111 MPa for cortical bone (Huang et al., 2019), 896 MPa for Titanium alloy (Ti-6Al-4V) (Athar et al., 2024), and 350–700 MPa for Medical stainless steel. Consequently, there were no secondary fractures or failures, confirming that all materials met strength specifications (Tables 1–3) (Figures 6A–C).

**TABLE 1:** Peak Von Mises stress of the tibia with TTT fixation in rats (MPa).

	3d	6d	9d	12d	30d
ACT	7.01	7.04	6.90	6.85	6.78
ATT	16.91	12.72	12.09	11.46	11.52
TPBT	9.40	9.36	9.38	9.35	9.30

ACT, axial compression test; ATT, axial torsion test; TPBT, three point bending test.

**TABLE 2:** Peak Von Mises stress of free bone fragments with TTT fixation in rats (MPa).

	3d	6d	9d	12d	30d
ACT	5.57	6.12	4.00	3.82	3.92
ATT	8.81	6.01	5.75	5.46	5.47
TPBT	4.37	3.43	3.61	3.33	3.36

ACT, axial compression test; ATT, axial torsion test; TPBT, three point bending test.

**TABLE 3:** Peak Von Mises stress of TTT fixation in rats (MPa).

	3d	6d	9d	12d	30d
ACT	7.29	7.73	7.26	7.07	7.24
ATT	26.19	19.18	18.14	17.17	17.25
TPBT	10.54	8.36	7.66	7.37	7.44

ACT, axial compression test; ATT, axial torsion test; TPBT, three point bending test.

### 3.3 Comparison of structural stability

Over a 30-day observation period (3 days–30 days post-surgery), the integration of transported bone fragments with surrounding bone tissue progressively influenced the tibial structural deformation across biomechanical tests. Quantitative measurements revealed time-dependent reductions in deformation: tibial deformation decreased by 15% in the axial compression test (0.020 mm–0.017 mm), 23.5% in the axial torsion test (from 0.068 mm–0.052 mm), and 19.4% in the three-point bending test (from 0.031 mm to 0.025 mm). The most significant reduction in deformation ranges from 13.23% (day6) to 25.00% (day12) in the axial torsion test, comparing to the changes in axial compression and three-point bending. These findings align with the theoretical relationship between deformation (δ) and structural stiffness (k), where δ = F/k—reduced deformation under constant experimental loads implies increased stiffness. The deformation trends indicate that mechanical stability enhancement paralleled morphological healing. (Table 4) (Figure 6D).

**TABLE 4:** Displacement deformation value of the overall structure of rat tibia after fixation with TTT fixation (mm).

	3d	6d	9d	12d	30d
ACT	0.020	0.019	0.018	0.017	0.017
ATT	0.068	0.059	0.054	0.051	0.052
TPBT	0.031	0.028	0.025	0.024	0.025

ACT, axial compression test; ATT, axial torsion test; TPBT, three point bending test.

## 4 Discussion

Diabetic foot ulcers (DFUs) impact a range of tissues—skin, nerves, blood vessels, muscles, tendons, and bones—necessitating advances in technology to repair and regenerate these structures. In 1989, Ilizarov introduced the “tension-stress” principle, establishing a theoretical foundation for using external fixators in orthopedics (Chen et al., 2020). Animal studies based on this principle showed that microvascular network regeneration in the distraction zone between bone ends precedes osteogenesis. Angiography results highlighted the reconstruction of neovascularization and microcirculation in the limb distraction area (Ilizarov, 1989a; Ilizarov, 1989b). Inspired by Ilizarov’s work on distraction osteogenesis, our team pioneered the use of the TTT technique to treat severe, refractory diabetic foot ulcers, achieving significant clinical outcomes. Previous research confirmed that TTT significantly improves DFU healing rates and reduces amputation and recurrence rates. Additionally, TTT enhances postoperative lower limb revascularization and foot microcirculation (Chen et al., 2022; Chen et al., 2020). Furthermore, our studies suggest that unilateral TTT promotes healing in bilateral DFUs, introducing a novel therapeutic approach for multifocal DFUs (Nie et al., 2021; Qin et al., 2023). Despite these promising results, the mechanisms behind TTT’s effectiveness in treating diabetic foot ulcers require further exploration.

TTT has emerged as an innovative method for treating chronic limb ischemic diseases, demonstrating significant clinical efficacy in DFU treatment. Peripheral arterial disease, caused by vascular occlusion, is a key factor in the persistence of non-healing DFUs (Meloni and Vas, 2025; Soyoye et al., 2021). In the early stages, bone transport surgery enhances blood supply by fostering the regeneration of microvascular networks in the lower limbs, crucial for healing ulcerative wounds. The local reparative effect of bone transport is crucial to its therapeutic success. Our research has further validated TTT’s efficacy and safety in treating DFUs through both retrospective single-center and prospective multicenter cohort studies (Chen et al., 2020). Although Ilizarov’s TTT technique showed increased angiogenesis at local bone transport sites in canine models, the underlying biological mechanisms remain unclear (Yang et al., 2022). Consequently, developing a substantive TTT animal model was necessary to deepen our understanding of these mechanisms. Our research team developed this model in diabetic foot rats, closely replicating clinical treatment protocols and outcomes. Our findings underscore the therapeutic potential of TTT in diabetic foot rats, particularly in enhancing angiogenesis and reducing inflammation at the wound site. These results reflect clinical efficacy and identify critical initiation times post-surgery. This timeframe aligns with clinical data, suggesting an especially active intrinsic mechanism of TTT that promotes wound healing. This suggests that the duration of bone transport is crucial for achieving TTT’s therapeutic effects. In our study, we meticulously established the diabetic rat TTT model and demonstrated that TTT promotes DFU healing by modulating angiogenesis and inflammatory pathways. This model more accurately replicates TTT’s clinical application in DFU patients with limb ischemia, surpassing previous research in scope (Yang et al., 2022).

Compared to previous studies in canine and rabbit models, our rat model offers higher throughput and genetic relevance to diabetic complications (Yang et al., 2022). Prior FEA studies in larger animals have reported similar trends in stress distribution and healing, supporting the validity of our model. However, our FEA approach assumes isotropic and homogeneous bone material properties, which may not fully capture the complex anisotropy of real bone tissue. Future studies incorporating patient-specific or region-specific material parameters are warranted. Importantly, this model can guide the optimization of TTT devices. By identifying safe load thresholds and fixation stress distributions, this model can inform clinical optimization of TTT device stiffness, screw placement, and transport protocols to minimize complications in DFU patients.

In this study, we developed a diabetic rat tibial transverse bone transport model that combines biomechanical principles with structural engineering concepts. We conducted an in-depth analysis of the tibial structure’s biomechanical properties using detailed three-dimensional simulations. The model showed high safety and stability, offering crucial mechanical guidance for clinical studies on tibial external fixation surgery.Von Mises stress was selected as the principal safety indicator because it reliably reflects multi-axial stress states in bone; while alternative criteria (e.g., fatigue, microcrack formation) may provide complementary insights, they require long-term *in vivo* validation beyond the scope of this study.

Unlike rabbit and canine models, which allow larger fixation devices and higher loading, the rat model provides higher throughput and stronger genetic relevance to diabetic complications. Despite scale differences, the stress distribution trends observed here are consistent with those reported in larger animal FEA models.

Although this study provides valuable insights, it has limitations. First, computational boundary conditions and loadings were simplified and may not reflect the full physiological complexity. The model assumes isotropic, homogeneous, and linear elastic bone properties. Second, the number of rats scanned was limited, and no *in vivo* biomechanical testing was performed for validation. Third, the 30-day observation period may not capture the complete remodeling and long-term outcomes. These limitations warrant further experimental and clinical investigations.

## 5 Conclusion

In conclusion, this finite element analysis supports the biomechanical safety and stability of the TTT rat model. However, further experimental and clinical validation is necessary before widespread application. Moreover, the research highlights the potential of this technique in treating ischemic diseases of the lower limbs, particularly diabetic foot ulcers. Our findings provide valuable insights into the role of mechanical stimulation in vascular regeneration and tissue repair, pointing the way forward for future research and clinical applications. These insights encourage further in-depth studies of this technology to optimize treatment strategies and improve outcomes for diabetic foot and other similar conditions.

## Data Availability

The original contributions presented in the study are included in the article/supplementary material, further inquiries can be directed to the corresponding authors.
